# An autonomy-based approach to assisted suicide: a way to avoid the expressivist objection against assisted dying laws

**DOI:** 10.1136/jme-2022-108375

**Published:** 2022-09-07

**Authors:** Esther Braun

**Affiliations:** Institute for Medical Ethics and History of Medicine, Ruhr University Bochum, Bochum, Germany

**Keywords:** Autonomy, Euthanasia, Death, Ethics- Medical, Right to Die

## Abstract

In several jurisdictions, irremediable suffering from a medical condition is a legal requirement for access to assisted dying. According to the expressivist objection, allowing assisted dying for a specific group of persons, such as those with irremediable medical conditions, expresses the judgment that their lives are not worth living. While the expressivist objection has often been used to argue that assisted dying should not be legalised, I show that there is an alternative solution available to its proponents. An autonomy-based approach to assisted suicide regards the provision of assisted suicide (but not euthanasia) as justified when it is autonomously requested by a person, irrespective of whether this is in her best interests. Such an approach has been put forward by a recent judgment of the German Federal Constitutional Court, which understands assisted suicide as an expression of the person’s right to a self-determined death. It does not allow for beneficence-based restrictions regarding the person’s suffering or medical diagnosis and therefore avoids the expressivist objection. I argue that on an autonomy-based approach, assisted suicide should not be understood as a medical procedure but rather as the person’s autonomous action.

## Introduction

In recent years, an increasing number of countries have legalised some forms of assisted dying (ie, assisted suicide and/or euthanasia). In several of these countries, irremediable suffering from a medical condition is a necessary legal criterion for access to assisted dying. This criterion corresponds to the ethical argument that assisted dying is justified partly because it relieves irremediable suffering and can thus be in the person’s best interests. Another element of the ethical justification of assisted dying refers to the value of making an autonomous decision about the circumstances of one’s own death. Correspondingly, autonomy of the request—that is, decision-making capacity and voluntariness—is also a legal requirement for access to assisted dying in many jurisdictions.^1^ However, autonomy is not usually regarded as sufficient for the ethical or legal permissibility of assisted dying.

In contrast, a recent judgment of the German Federal Constitutional Court justifies assisted suicide—but not euthanasia—on the basis of autonomy and declares that assisted suicide is an expression of the right to a self-determined death. This autonomy-based approach does not allow for restrictions regarding the person’s suffering or medical diagnosis.

According to the expressivist objection, which has recently received increasing attention in the literature,^2–6^ allowing assisted dying for only a specific group of persons—such as those with irremediable medical diseases or disabilities—expresses the disrespectful judgment that their lives are not worth living. In this article, I argue that if the expressivist objection is plausible, it only pertains to assisted dying laws that require beneficence-based eligibility criteria, such as suffering from an irremediable medical condition. The autonomy-based approach, however, avoids the expressivist objection.

The focus of this paper is not to examine whether the expressivist objection is valid. My aim is rather to demonstrate that endorsing the expressivist objection does not have to lead to the conclusion that assisted dying should be prohibited. To this end, I first explain the ethical foundations of the irremediable suffering requirement. I give an account of the expressivist objection and demonstrate how it may apply to assisted dying laws that require irremediable suffering. I then describe the autonomy-based approach to assisted suicide and show how it avoids the expressivist objection. Finally, I demonstrate that on the autonomy-based approach, assisted suicide should not be understood as a medical procedure but rather as the person’s autonomous action, and therefore does not have to follow traditional medical principles such as beneficence.

## The ‘irremediable suffering’ requirement

The ethical justification for assisted dying commonly refers to two principles: autonomy and beneficence. Based on the principle of autonomy, it is often argued that assisted dying is permissible because it allows the patient to autonomously control the circumstances of her own death. In reference to the principle of beneficence or compassion, it is argued that providing assisted dying can be in a patient’s best interests because it alleviates and prevents suffering.^7–11^

Following from this autonomy- and beneficence-based justification, proponents of the legalisation of assisted dying do not usually assume that providing assisted dying is *always* ethically justified, but rather that it is only justified under the conditions that the request is autonomous, and providing assisted dying is in the person’s best interests.^1^

It is generally assumed that the latter condition is fulfilled when the person is suffering severely from an irremediable medical condition (ie, a disease or disability), which is a necessary legal requirement for assisted dying in many jurisdictions, such as the Netherlands, Belgium, Luxembourg and Canada.^1^

In the Netherlands, where assisted dying has been legal for several decades, alleviating the patient’s suffering constitutes its primary legal justification. There, providing assisted dying is only justified when a physician’s duty to preserve life conflicts with her duty to relieve suffering, which allows the physician to invoke *force majeure*. The patient’s suffering must be unbearable without prospect of improvement and without reasonable alternatives to relieve suffering.^12 13^

Consequently, such a beneficence-based argument assumes that death can be objectively good for the person who requests assisted dying. This can be explained in reference to the deprivation view of death, a popular account of why death can be bad for someone. According to the deprivation view, death is bad for a person if it deprives her of a future that contains a positive net amount of well-being.^14^ Conversely, death is good for a person if it prevents a future that would have contained a negative net amount of well-being. To determine whether death would be good or bad for a person, we must make predictions about how the person’s future would likely go. A (partly) beneficence-based approach to assisted dying therefore requires an objective judgment of the person’s suffering and how likely it is to persist indefinitely.

In the Netherlands, the severity of the patient’s suffering must be evaluated by a physician.^15^ The physician must also make an objective judgment of whether the person’s suffering is irremediable, that is, whether there are other viable options available to treat the patient’s medical condition. Persons who do not fulfil this requirement, such as those ‘tired of life’, are therefore not eligible for assisted dying under the Dutch law.^12^

There is, thus, an element of indirect paternalism present in assisted dying laws that require irremediable suffering.^16^ The requirement aims to ensure that assisted dying is not available to persons for whom this would not be an objective benefit,^17^ even if they make an autonomous request.

## The expressivist objection

According to the expressivist objection, allowing assisted dying for a specific group of persons (eg, those who are judged to be suffering irremediably from a disease or disability) expresses the judgment that their lives are not worth living.

A version of the expressivist argument has been prominent in the debate on prenatal genetic diagnosis. There, it has been argued that restricting prenatal genetic testing to clinically ‘severe’ conditions sends a negative message to persons affected by those conditions.^18^ If only testing for particular conditions is allowed, this makes implicit normative judgments about which kinds of conditions and disabilities justify termination of a pregnancy.^19^

Reed has recently argued that the expressivist objection is more powerful in reference to assisted dying than in reference to prenatal diagnosis, even though the former has received less attention. He argues that if assisted dying is legally allowed only for patients who are suffering from irremediable or terminal illnesses or disabilities, this expresses the judgment that a life with such conditions is not worth living.^2^

As the above discussion of the irremediable suffering requirement has shown, justifying assisted dying (partly) in reference to beneficence entails the judgment that some lives are not worth living due to the amount of suffering they contain. The beneficence-based argument assumes that if a person suffers unbearably and the suffering is expected to continue in the future, it can be in her best interests to die.

Judging a person’s life as not worth living because she is suffering from an irremediable medical condition may contain an implicit judgment about the value of the lives of other persons with the same condition. As Kim states it: “If we justify ending the life […] of a person with D (a disability) because an absence of a life with D is better than a life with D, then that is a judgment that life with D is not worth living.”^3^ Or, according to Reed: “when we allow PAS [physician-assisted suicide] for individuals who are terminally ill or facing some severe disease or disability, we send a message of disrespect to all individuals who face such adversities in that we imply that they are inferior or their lives are not worth living […] precisely insofar as they are diseased or disabled.”^2^

A possible reply to the expressivist objection is that in choosing assisted dying, the requestor only makes an individual, private judgment about the value of her own life.^3 4^ For example, Colburn claims that the expressivist objection is mistaken because it fails to acknowledge the argument for assisted dying, which is ‘not that some lives are less worth living than others, but rather that each individual must decide what makes their life worth living’.^20^

However, if the judgment of whether one’s life is worth living were up to each individual, the decision for or against assisted dying should indeed be up to *each* individual—and not only to those individuals who are judged to be suffering severely enough.^5^ Assisted dying laws that require irremediable suffering allow individuals who autonomously wish to request assisted suicide to do so *only if* they have an irremediable disease or disability. Such a restriction implies that some persons—those with certain medical conditions or disabilities—have understandable reasons for wanting to die while others do not.

In the Netherlands, the law requires that doctor and patient must decide *together* that there is no reasonable alternative to assisted dying. Kim states that this requirement ‘really is a value judgment: not living is a better option than any alternative life’ with the condition the person is suffering from. The requestor is thus asking the doctor ‘to affirm the requestor’s judgment that his or her life is not worth living and to act on that shared judgment’.^3^

There may be other possible objections against the expressivist argument—however, I will not explore these in the context of this paper and, for the sake of the argument, assume that the expressivist objection is valid. My aim in the following section is rather to challenge the practical conclusion that is usually drawn from the expressivist objection.

## An autonomy-based approach

According to many proponents of the expressivist objection, the evaluative judgments expressed by allowing assisted dying for certain groups of persons have such detrimental effects for the societal view of these groups that as a consequence, assisted dying should not be legalised at all.^4^ But this need not be the case.

In the context of prenatal genetic diagnosis, some proponents of the expressivist objection endorse an unrestricted testing policy that does not distinguish disabilities from other non-medical traits that might be relevant to prospective parents. If the state adopts such a policy based on procreative autonomy, it remains strictly neutral about ‘what kinds of children should and should not be brought into the world’.^18^ A structurally similar solution is available for the context of assisted dying.

In an important judgment from 2020, the German Federal Constitutional Court put forward a justification of assisted suicide—but not euthanasia, which remains illegal in Germany—based on autonomy.^13^ It declared a ‘right to a self-determined death’, rendering a law established in 2015 that criminalised professional assisted suicide services (*geschäftsmäßige Sterbehilfe*) unconstitutional.^21^ While the concrete legal regulation of assisted suicide in Germany is currently still outstanding, the Constitutional Court binds the legal framework to some requirements that will arguably make it ‘the most liberal one in the world’.^22^

The judgment states that the constitutional right of personality entails the right to end one’s own life and to seek and make use of assistance from third parties for this purpose. It stresses that the decision to end one’s own life and the reasons for this decision are highly personal. Decisions for assisted dying that are based on individual definitions of ‘quality of life and a meaningful existence’^21^ ^2^ (para. 210) must be respected as an act of personal self-determination. Objective judgments of an individual’s subjective reasons for ending her life are impermissible—therefore, access to assisted suicide cannot be limited to persons with certain medical conditions. The Court states:

The right to a self-determined death, as an expression of personal freedom, is not limited to situations defined by external causes. The right to determine one’s own life […] is in particular not limited to serious or incurable illness, nor does it apply only in certain stages of life or illness.”^21^ (para. 210)

Although the judgment declares a *right* to a self-determined death, this does not entail a moral or legal *obligation* to accede to requests for assisted suicide.^23^ The judgment stresses that no one can be obliged to provide assisted suicide^21^ (para. 342). It is a different question how many people would be *willing* to provide assisted suicide based only on an autonomous request—the autonomy-based approach merely argues that it should be legal to do so.

The judgment suggests some restrictions of access to assisted suicide. However, these do not pertain to the person’s suffering or any other objective reasons for her wish to die but rather aim to ensure that the request is autonomous. Corresponding to informed consent standards in the context of curative medical treatment, the person must have decision-making capacity, the decision must be informed and voluntary, and be based on a ‘certain internal stability’ (para. 244).

An autonomy-based approach does not allow for objective judgments of whether assisted suicide would be in the requestor’s best interests. Since all permissible legal restrictions refer to autonomy, the law does not express any judgments about whether lives with certain diseases or disabilities are worth living. Instead, the judgment of whether her life is worth living is strictly the patient’s. The autonomy-based approach thus avoids the expressivist objection.

On an autonomy-based approach, it is legal to provide assisted suicide to anyone who makes an autonomous request, even if there would have been options available to improve the person’s medical condition or if she is not suffering from a medical condition at all. This includes, for example, persons ‘tired of living’ without an irremediable medical disease. Autonomy-based legislation does not assume that some forms of suffering justify ending one’s life while others do not.

In objection to this, it could be argued that the autonomy-based approach does not fully avoid the expressivist objection because beneficence-based reasons may still play a role in the assessment of decision-making capacity. Decision-making capacity can be understood as risk relative, meaning that riskier choices require a higher threshold for capacity.^24^ If setting the threshold for capacity involves a judgment of how risky and therefore, how potentially harmful a particular person’s choice for assisted suicide is, assessing capacity would still entail an evaluative judgment of the severity of the person’s suffering and the value of her life.

Whether the threshold for decision-making capacity should be risk relative is, however, highly controversial.^24 25^ On an autonomy-based approach, the motives of a choice for assisted dying should not be judged according to standards of ‘objective rationality’^21^ (para. 210). Therefore, setting the threshold for capacity based on objective risks and harms does not seem adequate. If the autonomy-based approach consistently requires a high standard for capacity in the context of assisted suicide without assessing the potential harms of a particular individual’s choice, it still avoids the expressivist objection. This implies that all reasons for wanting to end one’s life should be acceptable, and that the standard for decision-making capacity should not be lower for those who are thought to objectively benefit from assisted suicide.

Second, it might be objected that even under an autonomy-based legislative framework, individual providers could still believe that the life of a person with a disease or disability who requests assisted dying is not worth living. While this may be the case, the opinions of individual persons differ significantly from judgments expressed in the law, since laws express collective moral norms. When a law—as a public act of the state—expresses a certain judgment, this has greater force than a private individual choice.^2 6^ The expressivist objection is therefore primarily directed at public legal norms, not at individual actions.^6^

It is likely that under autonomy-based legislation, most requests for assisted suicide will be from people with terminal or irremediable diseases or disabilities. However, the private decisions of these persons, and their individual judgments of whether their lives are worth living, would not have any legal or public endorsement.

## Implications of the autonomy-based approach

Assisted dying is often understood as a kind of medical practice. As stated above, the Dutch legal justification of assisted dying explicitly refers to *physicians’* duties, and an incurable medical condition is a legal eligibility criterion in several jurisdictions.^1^ Similarly, Sumner argues that ‘assisted death is a form of medical treatment and, as such, should be reserved for the relief of suffering due to a medical condition’.^9^ Normally, medical treatments are only provided when there is medical benefit to the patient—for example, we do not provide futile medical treatments just because a patient autonomously requests them. If assisted dying is treated as a medical practice, it could be assumed that as with other medical treatments, it can be *indicated* in cases where the criterion of ‘irremediable suffering’ is fulfilled because it would benefit the patient. Jansen *et al* assume, too, that assisted dying is a ‘medical practice’ and believe that it should follow general principles for medical interventions that are carried out by physicians.^17^ They argue that allowing physicians to provide assisted dying when this is not in patients’ ‘best medical interests’ would lead to a harmful change of the physician’s social role.^3^

However, this argument does not apply to an autonomy-based approach. On such an approach, assisted suicide should not be understood as a medical procedure that can be ‘indicated’ but rather as an autonomous action that can be carried out for all sorts of reasons that should not be judged by others. Since a medical condition is not a precondition for access to assisted suicide on this approach, it does not necessarily require the expertise and participation of a physician.^13^ If we do not understand assisted suicide as a medical practice, beneficence-based standards for medical interventions do not have to be followed. Therefore, adopting an autonomy-based approach to assisted suicide does not lead to abandoning established principles of the medical profession.

In countries that follow a more beneficence-based approach, such as the Netherlands, assisted dying is much more often carried out as euthanasia.^26^ In contrast, the judgment of the German Federal Constitutional Court which puts forward an autonomy-based approach only concerns assisted suicide, while euthanasia remains illegal. This restriction to actions carried out by the person *herself*, rather than by a physician, emphasises the understanding of assisted suicide as the patient’s autonomous action instead of a medical procedure.^13^

It could be argued that an approach that only allows assisted suicide but not euthanasia would be discriminatory towards those who cannot self-administer lethal medication. However, it seems that all who can communicate an autonomous request for assisted dying can also self-administer medication in some way—even if this may require technically sophisticated means, such as controlling a lethal infusion with one’s eye movements.^27^ The autonomy-based approach stresses that it should be the person herself who carries out the action that ends her life, and not a third party—however, it does allow support in carrying out this action.

Adopting the autonomy-based approach to assisted dying does not necessarily result in having to permit all other self-regarding actions based on autonomy, such as using addictive drugs or selling one’s organs. While we typically assume that individuals are free to harm *themselves*, potential harm to *others* can justify limiting individual freedom.^28^ When considering whether to legally permit any potentially harmful self-regarding action, we also take the harms to others and society that permitting the action would cause into account. The respective harms of such actions would have to be assessed in each case.

With respect to assisted dying, it might be argued that an autonomy-based approach to assisted suicide leads to more significant harms than a restriction of assisted dying to those who are judged to be suffering irremediably. It is beyond the scope of this paper to fully evaluate the practical consequences of an autonomy-based approach in comparison with a beneficence-based approach. However, the article has shown that an autonomy-based approach avoids the expressivist objection against assisted dying laws, and therefore at least avoids some potential societal harms that could arise from legalising assisted dying.

## Conclusion

Assuming that the expressivist argument is valid, it only applies to (partly) beneficence-based approaches to assisted dying that require irremediable suffering. An autonomy-based approach to assisted suicide, as put forward by the German Federal Constitutional Court, avoids the expressivist objection. It understands assisted suicide as an act justified by autonomy and does not imply objective judgments of whether the person’s life is worth living. I have argued that on an autonomy-based approach, assisted suicide should not be understood as a medical intervention but rather as an autonomous action that does not invoke traditional medical principles such as beneficence.

## Data Availability

Data sharing not applicable as no datasets generated and/or analysed for this study. Not applicable.

## Footnotes

The best interests requirement is not based on a fully subjective account of well-being that understands benefit to a patient as equivalent with the fulfilment of her autonomous preferences.^11^ If that were the case, the second condition would be redundant and the autonomy- and beneficence-based approach would collapse into a purely autonomy-based approach.^29^

All citations refer to the official English translation of the judgment.

Note, however, that it is controversial whether objective medical benefit to the patient is necessary for the permissibility of medical interventions. For example, interventions such as some plastic surgeries or vasectomies are not necessarily in the patient’s objective best medical interests.^29 30^
